# 5-Chloro-3-cyclo­hexyl­sulfinyl-2-methyl-1-benzofuran

**DOI:** 10.1107/S1600536811007859

**Published:** 2011-03-09

**Authors:** Hong Dae Choi, Pil Ja Seo, Byeng Wha Son, Uk Lee

**Affiliations:** a Department of Chemistry, Dongeui University, San 24 Kaya-dong Busanjin-gu, Busan 614-714, Republic of Korea; b Department of Chemistry, Pukyong National University, 599-1 Daeyeon 3-dong, Nam-gu, Busan 608-737, Republic of Korea

## Abstract

There are two independent mol­ecules in the asymmetric unit of the title compound, C_15_H_17_ClO_2_S, in each of which the cyclo­hexyl rings adopt chair conformations. In the crystal, mol­ecules are linked by weak inter­molecular C—H⋯O hydrogen bonds.

## Related literature

For the pharmacological activity of benzofuran compounds, see: Aslam *et al.* (2006 ▶); Galal *et al.* (2009 ▶); Khan *et al.* (2005 ▶). For natural products with benzofuran rings, see: Akgul & Anil (2003 ▶); Soekamto *et al.* (2003 ▶). For the structure of 5-bromo-3-cyclo­hexyl­sulfinyl-2-methyl-1-benzofuran, see: Choi *et al.* (2011 ▶).
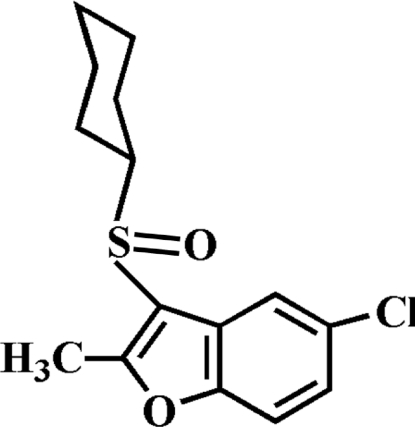


## Experimental

### 

#### Crystal data

C_15_H_17_ClO_2_S
*M* *_r_* = 296.80
Monoclinic, 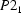

*a* = 12.0755 (2) Å
*b* = 9.0033 (2) Å
*c* = 13.9112 (2) Å
β = 108.667 (1)°
*V* = 1432.86 (4) Å^3^
*Z* = 4
Mo *K*α radiation
μ = 0.41 mm^−1^
*T* = 173 K
0.28 × 0.24 × 0.17 mm

#### Data collection

Bruker SMART APEXII CCD diffractometer
Absorption correction: multi-scan (*SADABS*; Bruker, 2009 ▶) *T*_min_ = 0.896, *T*_max_ = 0.932
14184 measured reflections
6583 independent reflections
6143 reflections with *I* > 2σ(*I*)
*R*_int_ = 0.026

#### Refinement

*R*[*F* ^2^ > 2σ(*F* ^2^)] = 0.033
*wR*(*F* ^2^) = 0.084
*S* = 1.03
6583 reflections
345 parameters
1 restraint
H-atom parameters constrained
Δρ_max_ = 0.40 e Å^−3^
Δρ_min_ = −0.37 e Å^−3^
Absolute structure: Flack (1983 ▶), 2822 Friedel pairs
Flack parameter: 0.03 (4)

### 

Data collection: *APEX2* (Bruker, 2009 ▶); cell refinement: *SAINT* (Bruker, 2009 ▶); data reduction: *SAINT*; program(s) used to solve structure: *SHELXS97* (Sheldrick, 2008 ▶); program(s) used to refine structure: *SHELXL97* (Sheldrick, 2008 ▶); molecular graphics: *ORTEP-3* (Farrugia, 1997 ▶) and *DIAMOND* (Brandenburg, 1998 ▶); software used to prepare material for publication: *SHELXL97*.

## Supplementary Material

Crystal structure: contains datablocks I. DOI: 10.1107/S1600536811007859/hg5004sup1.cif

Structure factors: contains datablocks I. DOI: 10.1107/S1600536811007859/hg5004Isup2.hkl

Additional supplementary materials:  crystallographic information; 3D view; checkCIF report

## Figures and Tables

**Table 1:** Hydrogen-bond geometry (Å, °)

*D*—H⋯*A*	*D*—H	H⋯*A*	*D*⋯*A*	*D*—H⋯*A*
C5—H5⋯O2^i^	0.95	2.54	3.469 (3)	166

Symmetry code: (i) 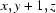.

**Fig. 1.**
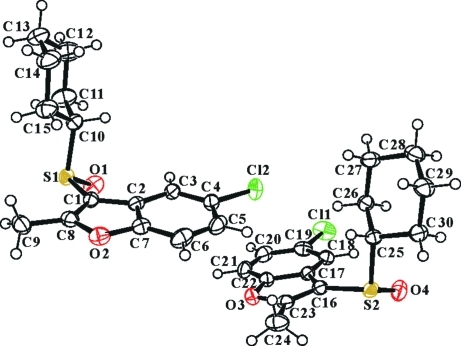
The molecular structure of the title compound with the atom numbering scheme. Displacement ellipsoids are drawn at the 50% probability level. H atoms are presented as a small spheres of arbitrary radius.

## supplementary crystallographic information

### Comment

Many compounds containing a benzofuran ring exhibit interesting pharmacological properties such as antifungal, antitumor and antiviral, and antimicrobial activities (Aslam *et al.*, 2006, Galal *et al.*, 2009, Khan *et al.*, 2005). These compounds occur in a wide range of natural products (Akgul & Anil, 2003; Soekamto *et al.*, 2003). As a part of our ongoing study of the substituent effect on the solid state structures of 3-cyclohexylsulfinyl-5-halo-2-methyl-1-benzofuran analogues (Choi *et al.*, 2011), we report herein on the crystal structure of the title compound.

The title compound crystallizes as the non-centrosymmetric space group *P*2_1_ in spite of having no asymmetric C atoms. The asymmetric unit of the title compound is shown in Fig. 1. There are two independent unique molecules [ A & B] in which the benzofuran unit is essentially planar, with a mean deviation of 0.007 (1) Å for A and 0.009 (1) Å for B, respectively, from the least-squares plane defined by the nine constituent atoms. The cyclohexyl rings are in the chair form. The molecular packing  is stabilized by weak intermolecular C—H···O hydrogen bonds between a benzene H atom and the O atom of the sulfinyl group (Table 1; C5—H5···O2^i^).

### Experimental

77% 3-chloroperoxybenzoic acid (269 mg, 1.2 mmol) was added in small portions to a stirred solution of 5-chloro-3-cyclohexylsulfanyl-2-methyl-1-benzofuran (309 mg, 1.1 mmol) in dichloromethane (40 mL) at 273 K. After being stirred at room temperature for 4h, the mixture was washed with saturated sodium bicarbonate solution and the organic layer was separated, dried over magnesium sulfate, filtered and concentrated at reduced pressure. The residue was purified by column chromatography (hexane–ethyl acetate, 2:1 v/v) to afford the title compound as a colorless solid [yield 76%, m.p. 382–383 K; *R*_f_ = 0.47 (hexane–ethyl acetate, 2:1 v/v)]. Single crystals suitable for X-ray diffraction were prepared by slow evaporation of a solution of the title compound in aectone at room temperature.

### Refinement

All H atoms were positioned geometrically and refined using a riding model, with C—H = 0.95 Å  for aryl, 1.00 Å   for methine, 0.99 Å for methylene and 0.98 Å   for methyl H atoms, respectively. *U*_iso_(H) =1.2*U*_eq_(C)  for aryl,  methine and methylene, and 1.5*U*_eq_(C) for methyl H atoms.

### Crystal data

**Table:**

C_15_H_17_ClO_2_S	*F*(000) = 624
*M**_r_* = 296.80	*D*_x_ = 1.376 Mg m^−^^3^
Monoclinic, *P*2_1_	Mo *K*α radiation, λ = 0.71073 Å
Hall symbol: P 2yb	Cell parameters from 6118 reflections
*a* = 12.0755 (2) Å	θ = 2.7–28.0°
*b* = 9.0033 (2) Å	µ = 0.41 mm^−^^1^
*c* = 13.9112 (2) Å	*T* = 173 K
β = 108.667 (1)°	Block, colourless
*V* = 1432.86 (4) Å^3^	0.28 × 0.24 × 0.17 mm
*Z* = 4	

### Data collection

**Table:**

Bruker SMART APEXII CCD diffractometer	6583 independent reflections
Radiation source: rotating anode	6143 reflections with *I* > 2σ(*I*)
graphite multilayer	*R*_int_ = 0.026
Detector resolution: 10.0 pixels mm^-1^	θ_max_ = 28.2°, θ_min_ = 1.6°
φ and ω scans	*h* = −12→16
Absorption correction: multi-scan (*SADABS*; Bruker, 2009)	*k* = −11→11
*T*_min_ = 0.896, *T*_max_ = 0.932	*l* = −18→16
14184 measured reflections	

### Refinement

**Table:**

Refinement on *F*^2^	Secondary atom site location: difference Fourier map
Least-squares matrix: full	Hydrogen site location: difference Fourier map
*R*[*F*^2^ > 2σ(*F*^2^)] = 0.033	H-atom parameters constrained
*wR*(*F*^2^) = 0.084	*w* = 1/[σ^2^(*F*_o_^2^) + (0.0466*P*)^2^ + 0.1747*P*] where *P* = (*F*_o_^2^ + 2*F*_c_^2^)/3
*S* = 1.03	(Δ/σ)_max_ = 0.001
6583 reflections	Δρ_max_ = 0.40 e Å^−^^3^
345 parameters	Δρ_min_ = −0.37 e Å^−^^3^
1 restraint	Absolute structure: Flack (1983), 2822 Friedel pairs
Primary atom site location: structure-invariant direct methods	Flack parameter: 0.03 (4)

### Special details

**Table:**

Geometry. All esds (except the esd in the dihedral angle between two l.s. planes) are estimated using the full covariance matrix. The cell esds are taken into account individually in the estimation of esds in distances, angles and torsion angles; correlations between esds in cell parameters are only used when they are defined by crystal symmetry. An approximate (isotropic) treatment of cell esds is used for estimating esds involving l.s. planes.
Refinement. Refinement of F^2^ against ALL reflections. The weighted R-factor wR and goodness of fit S are based on F^2^, conventional R-factors R are based on F, with F set to zero for negative F^2^. The threshold expression of F^2^ > 2sigma(F^2^) is used only for calculating R-factors(gt) etc. and is not relevant to the choice of reflections for refinement. R-factors based on F^2^ are statistically about twice as large as those based on F, and R- factors based on ALL data will be even larger.

### Fractional atomic coordinates and isotropic or equivalent isotropic displacement parameters (Å^2^)

**Table:**

	*x*	*y*	*z*	*U*_iso_*/*U*_eq_	
S1	0.16903 (4)	−0.32206 (5)	0.81418 (3)	0.02606 (10)	
S2	0.64550 (4)	0.64961 (5)	0.83739 (3)	0.02826 (10)	
Cl1	0.89697 (5)	0.15052 (8)	0.68607 (4)	0.04851 (15)	
Cl2	0.38281 (5)	0.23270 (7)	0.69511 (4)	0.04830 (15)	
O1	0.09876 (12)	0.04307 (17)	0.93970 (10)	0.0328 (3)	
O2	0.28828 (11)	−0.33014 (19)	0.80366 (11)	0.0378 (3)	
O3	0.60633 (12)	0.25359 (17)	0.94106 (9)	0.0301 (3)	
O4	0.75245 (12)	0.67789 (19)	0.80949 (11)	0.0394 (3)	
C25	0.52029 (14)	0.6537 (2)	0.72146 (12)	0.0245 (3)	
H25	0.4493	0.6236	0.7389	0.029*	
C1	0.15225 (15)	−0.1419 (2)	0.85605 (12)	0.0232 (4)	
C2	0.19911 (16)	−0.0065 (2)	0.83065 (12)	0.0231 (4)	
C3	0.26704 (16)	0.0312 (2)	0.76970 (13)	0.0265 (4)	
H3	0.2921	−0.0413	0.7315	0.032*	
C4	0.29587 (16)	0.1790 (3)	0.76777 (13)	0.0312 (4)	
C5	0.26014 (19)	0.2885 (2)	0.82263 (16)	0.0371 (5)	
H5	0.2820	0.3890	0.8184	0.045*	
C6	0.19328 (19)	0.2514 (2)	0.88290 (17)	0.0373 (5)	
H6	0.1685	0.3239	0.9212	0.045*	
C7	0.16438 (17)	0.1037 (2)	0.88456 (14)	0.0272 (4)	
C8	0.09347 (16)	−0.1064 (2)	0.92107 (13)	0.0274 (4)	
C9	0.02454 (18)	−0.1967 (3)	0.97107 (15)	0.0367 (5)	
H9A	0.0522	−0.2997	0.9773	0.055*	
H9B	−0.0583	−0.1936	0.9302	0.055*	
H9C	0.0343	−0.1563	1.0387	0.055*	
C10	0.06561 (15)	−0.3065 (2)	0.68610 (13)	0.0273 (4)	
H10	0.0837	−0.2148	0.6534	0.033*	
C11	0.0809 (2)	−0.4417 (3)	0.62566 (17)	0.0464 (6)	
H11A	0.0717	−0.5336	0.6614	0.056*	
H11B	0.1603	−0.4414	0.6195	0.056*	
C12	−0.0104 (3)	−0.4381 (4)	0.52011 (19)	0.0577 (7)	
H12A	0.0033	−0.3500	0.4828	0.069*	
H12B	−0.0017	−0.5277	0.4819	0.069*	
C13	−0.1333 (2)	−0.4322 (3)	0.52604 (18)	0.0524 (7)	
H13A	−0.1902	−0.4280	0.4567	0.063*	
H13B	−0.1490	−0.5234	0.5593	0.063*	
C14	−0.14857 (19)	−0.2984 (3)	0.58510 (17)	0.0507 (7)	
H14A	−0.2279	−0.2996	0.5914	0.061*	
H14B	−0.1411	−0.2073	0.5479	0.061*	
C15	−0.05747 (17)	−0.2951 (3)	0.69140 (15)	0.0415 (6)	
H15A	−0.0656	−0.2015	0.7259	0.050*	
H15B	−0.0720	−0.3788	0.7320	0.050*	
C16	0.64528 (16)	0.4596 (2)	0.86753 (13)	0.0252 (4)	
C17	0.70239 (16)	0.3378 (2)	0.83536 (13)	0.0240 (4)	
C18	0.77230 (16)	0.3203 (2)	0.77357 (13)	0.0272 (4)	
H18	0.7936	0.4025	0.7405	0.033*	
C19	0.80929 (15)	0.1776 (3)	0.76261 (13)	0.0315 (4)	
C20	0.77837 (19)	0.0540 (2)	0.80917 (16)	0.0358 (5)	
H20	0.8042	−0.0421	0.7979	0.043*	
C21	0.71070 (19)	0.0711 (2)	0.87124 (16)	0.0345 (5)	
H21	0.6897	−0.0113	0.9044	0.041*	
C22	0.67472 (16)	0.2129 (2)	0.88309 (13)	0.0279 (4)	
C23	0.59106 (17)	0.4040 (2)	0.93076 (13)	0.0283 (4)	
C24	0.52066 (19)	0.4726 (3)	0.98873 (15)	0.0405 (5)	
H24A	0.4389	0.4796	0.9455	0.061*	
H24B	0.5507	0.5723	1.0108	0.061*	
H24C	0.5258	0.4114	1.0482	0.061*	
C26	0.53191 (17)	0.5501 (2)	0.63839 (13)	0.0274 (4)	
H26A	0.5381	0.4460	0.6624	0.033*	
H26B	0.6038	0.5746	0.6220	0.033*	
C27	0.42505 (18)	0.5671 (3)	0.54340 (14)	0.0346 (5)	
H27A	0.4342	0.5024	0.4889	0.042*	
H27B	0.3542	0.5348	0.5588	0.042*	
C28	0.4101 (2)	0.7270 (3)	0.50687 (15)	0.0385 (5)	
H28A	0.4782	0.7570	0.4864	0.046*	
H28B	0.3391	0.7355	0.4468	0.046*	
C29	0.39932 (19)	0.8293 (3)	0.58943 (15)	0.0374 (5)	
H29A	0.3272	0.8048	0.6054	0.045*	
H29B	0.3927	0.9332	0.5649	0.045*	
C30	0.50495 (17)	0.8157 (2)	0.68588 (14)	0.0319 (4)	
H30A	0.4932	0.8792	0.7399	0.038*	
H30B	0.5763	0.8499	0.6720	0.038*	

### Atomic displacement parameters (Å^2^)

**Table:**

	*U*^11^	*U*^22^	*U*^33^	*U*^12^	*U*^13^	*U*^23^
S1	0.0268 (2)	0.0196 (2)	0.0307 (2)	0.00028 (18)	0.00769 (16)	0.00088 (17)
S2	0.0304 (2)	0.0215 (2)	0.0281 (2)	−0.00027 (18)	0.00275 (16)	−0.00124 (18)
Cl1	0.0433 (3)	0.0614 (4)	0.0446 (3)	0.0168 (3)	0.0194 (2)	−0.0047 (3)
Cl2	0.0514 (3)	0.0471 (3)	0.0473 (3)	−0.0212 (3)	0.0171 (2)	0.0081 (2)
O1	0.0322 (7)	0.0364 (8)	0.0313 (7)	0.0029 (6)	0.0121 (6)	−0.0073 (6)
O2	0.0282 (7)	0.0361 (8)	0.0478 (7)	0.0054 (7)	0.0102 (6)	−0.0047 (7)
O3	0.0315 (7)	0.0319 (8)	0.0278 (6)	−0.0040 (6)	0.0106 (5)	0.0038 (6)
O4	0.0300 (7)	0.0308 (8)	0.0520 (8)	−0.0038 (7)	0.0057 (6)	0.0082 (7)
C25	0.0213 (7)	0.0260 (9)	0.0248 (7)	0.0015 (7)	0.0055 (6)	−0.0004 (7)
C1	0.0213 (8)	0.0227 (9)	0.0246 (8)	0.0002 (7)	0.0058 (7)	0.0012 (7)
C2	0.0226 (9)	0.0206 (9)	0.0230 (8)	0.0006 (7)	0.0031 (7)	−0.0018 (6)
C3	0.0261 (9)	0.0253 (10)	0.0264 (8)	−0.0021 (7)	0.0059 (7)	0.0002 (7)
C4	0.0296 (9)	0.0308 (10)	0.0290 (8)	−0.0055 (9)	0.0036 (7)	0.0054 (8)
C5	0.0379 (12)	0.0201 (10)	0.0435 (11)	−0.0024 (8)	−0.0009 (10)	0.0018 (8)
C6	0.0363 (11)	0.0256 (11)	0.0448 (12)	0.0038 (9)	0.0055 (9)	−0.0095 (9)
C7	0.0251 (9)	0.0267 (10)	0.0277 (9)	0.0023 (7)	0.0054 (7)	−0.0025 (7)
C8	0.0253 (9)	0.0316 (10)	0.0236 (8)	0.0013 (8)	0.0056 (7)	0.0008 (7)
C9	0.0314 (10)	0.0496 (14)	0.0301 (9)	−0.0014 (10)	0.0113 (8)	0.0075 (9)
C10	0.0277 (9)	0.0253 (10)	0.0283 (8)	−0.0013 (7)	0.0083 (7)	−0.0033 (7)
C11	0.0475 (13)	0.0450 (14)	0.0436 (11)	0.0055 (11)	0.0099 (10)	−0.0172 (10)
C12	0.0657 (17)	0.0612 (18)	0.0415 (13)	0.0002 (14)	0.0105 (12)	−0.0215 (12)
C13	0.0524 (15)	0.0524 (16)	0.0398 (12)	−0.0197 (12)	−0.0031 (10)	−0.0041 (11)
C14	0.0266 (10)	0.073 (2)	0.0454 (12)	−0.0056 (11)	0.0022 (9)	−0.0035 (12)
C15	0.0268 (10)	0.0604 (17)	0.0372 (10)	−0.0027 (10)	0.0103 (8)	−0.0071 (10)
C16	0.0277 (9)	0.0224 (9)	0.0229 (8)	−0.0001 (7)	0.0045 (7)	−0.0010 (7)
C17	0.0239 (9)	0.0208 (9)	0.0242 (8)	−0.0004 (7)	0.0034 (7)	0.0006 (7)
C18	0.0262 (9)	0.0286 (10)	0.0248 (8)	0.0010 (8)	0.0055 (7)	0.0020 (7)
C19	0.0260 (8)	0.0382 (11)	0.0282 (8)	0.0071 (9)	0.0056 (7)	−0.0021 (9)
C20	0.0367 (12)	0.0236 (10)	0.0394 (11)	0.0055 (9)	0.0017 (9)	−0.0021 (8)
C21	0.0378 (12)	0.0220 (10)	0.0384 (10)	−0.0032 (8)	0.0050 (9)	0.0048 (8)
C22	0.0256 (9)	0.0305 (11)	0.0247 (8)	−0.0043 (8)	0.0041 (7)	0.0036 (7)
C23	0.0263 (9)	0.0321 (10)	0.0229 (8)	−0.0022 (8)	0.0029 (7)	−0.0027 (7)
C24	0.0330 (12)	0.0583 (16)	0.0319 (10)	−0.0009 (10)	0.0128 (8)	−0.0094 (10)
C26	0.0311 (10)	0.0235 (9)	0.0274 (8)	0.0008 (7)	0.0089 (7)	−0.0016 (7)
C27	0.0363 (11)	0.0354 (12)	0.0270 (9)	0.0011 (9)	0.0030 (8)	−0.0049 (8)
C28	0.0405 (12)	0.0407 (13)	0.0280 (9)	0.0041 (10)	0.0021 (8)	0.0046 (9)
C29	0.0369 (11)	0.0328 (12)	0.0362 (10)	0.0100 (9)	0.0029 (8)	0.0047 (9)
C30	0.0333 (10)	0.0243 (10)	0.0330 (9)	0.0055 (8)	0.0036 (8)	−0.0005 (8)

### Geometric parameters (Å, °)

**Table:**

S1—O2	1.4951 (13)	C12—H12B	0.9900
S1—C1	1.7569 (19)	C13—C14	1.502 (4)
S1—C10	1.8246 (17)	C13—H13A	0.9900
S2—O4	1.4847 (15)	C13—H13B	0.9900
S2—C16	1.762 (2)	C14—C15	1.535 (3)
S2—C25	1.8241 (16)	C14—H14A	0.9900
Cl1—C19	1.7424 (18)	C14—H14B	0.9900
Cl2—C4	1.7427 (19)	C15—H15A	0.9900
O1—C8	1.368 (3)	C15—H15B	0.9900
O1—C7	1.380 (2)	C16—C23	1.350 (3)
O3—C23	1.368 (3)	C16—C17	1.441 (3)
O3—C22	1.376 (2)	C17—C18	1.393 (3)
C25—C26	1.526 (2)	C17—C22	1.400 (3)
C25—C30	1.532 (3)	C18—C19	1.385 (3)
C25—H25	1.0000	C18—H18	0.9500
C1—C8	1.355 (2)	C19—C20	1.397 (3)
C1—C2	1.435 (3)	C20—C21	1.374 (3)
C2—C7	1.387 (3)	C20—H20	0.9500
C2—C3	1.397 (3)	C21—C22	1.376 (3)
C3—C4	1.377 (3)	C21—H21	0.9500
C3—H3	0.9500	C23—C24	1.481 (3)
C4—C5	1.397 (3)	C24—H24A	0.9800
C5—C6	1.378 (3)	C24—H24B	0.9800
C5—H5	0.9500	C24—H24C	0.9800
C6—C7	1.377 (3)	C26—C27	1.531 (2)
C6—H6	0.9500	C26—H26A	0.9900
C8—C9	1.486 (3)	C26—H26B	0.9900
C9—H9A	0.9800	C27—C28	1.518 (3)
C9—H9B	0.9800	C27—H27A	0.9900
C9—H9C	0.9800	C27—H27B	0.9900
C10—C15	1.515 (3)	C28—C29	1.510 (3)
C10—C11	1.523 (3)	C28—H28A	0.9900
C10—H10	1.0000	C28—H28B	0.9900
C11—C12	1.528 (3)	C29—C30	1.532 (3)
C11—H11A	0.9900	C29—H29A	0.9900
C11—H11B	0.9900	C29—H29B	0.9900
C12—C13	1.514 (4)	C30—H30A	0.9900
C12—H12A	0.9900	C30—H30B	0.9900
			
O2—S1—C1	107.11 (9)	C15—C14—H14A	109.3
O2—S1—C10	106.72 (8)	C13—C14—H14B	109.3
C1—S1—C10	97.91 (8)	C15—C14—H14B	109.3
O4—S2—C16	107.24 (9)	H14A—C14—H14B	108.0
O4—S2—C25	108.04 (8)	C10—C15—C14	111.32 (17)
C16—S2—C25	99.55 (9)	C10—C15—H15A	109.4
C8—O1—C7	106.52 (14)	C14—C15—H15A	109.4
C23—O3—C22	106.75 (14)	C10—C15—H15B	109.4
C26—C25—C30	111.59 (14)	C14—C15—H15B	109.4
C26—C25—S2	113.96 (12)	H15A—C15—H15B	108.0
C30—C25—S2	106.56 (13)	C23—C16—C17	107.50 (17)
C26—C25—H25	108.2	C23—C16—S2	123.58 (15)
C30—C25—H25	108.2	C17—C16—S2	128.89 (14)
S2—C25—H25	108.2	C18—C17—C22	119.04 (18)
C8—C1—C2	107.22 (17)	C18—C17—C16	136.28 (18)
C8—C1—S1	125.45 (15)	C22—C17—C16	104.67 (16)
C2—C1—S1	127.32 (13)	C19—C18—C17	116.97 (18)
C7—C2—C3	119.46 (18)	C19—C18—H18	121.5
C7—C2—C1	105.27 (16)	C17—C18—H18	121.5
C3—C2—C1	135.26 (17)	C18—C19—C20	122.98 (18)
C4—C3—C2	116.61 (18)	C18—C19—Cl1	118.53 (16)
C4—C3—H3	121.7	C20—C19—Cl1	118.49 (17)
C2—C3—H3	121.7	C21—C20—C19	120.23 (19)
C3—C4—C5	123.14 (18)	C21—C20—H20	119.9
C3—C4—Cl2	118.84 (16)	C19—C20—H20	119.9
C5—C4—Cl2	118.02 (17)	C20—C21—C22	116.94 (19)
C6—C5—C4	120.3 (2)	C20—C21—H21	121.5
C6—C5—H5	119.8	C22—C21—H21	121.5
C4—C5—H5	119.8	C21—C22—O3	126.10 (17)
C7—C6—C5	116.4 (2)	C21—C22—C17	123.81 (18)
C7—C6—H6	121.8	O3—C22—C17	110.08 (17)
C5—C6—H6	121.8	C16—C23—O3	110.98 (17)
C6—C7—O1	125.83 (18)	C16—C23—C24	133.1 (2)
C6—C7—C2	124.0 (2)	O3—C23—C24	115.93 (18)
O1—C7—C2	110.13 (17)	C23—C24—H24A	109.5
C1—C8—O1	110.86 (17)	C23—C24—H24B	109.5
C1—C8—C9	132.5 (2)	H24A—C24—H24B	109.5
O1—C8—C9	116.66 (17)	C23—C24—H24C	109.5
C8—C9—H9A	109.5	H24A—C24—H24C	109.5
C8—C9—H9B	109.5	H24B—C24—H24C	109.5
H9A—C9—H9B	109.5	C25—C26—C27	109.65 (15)
C8—C9—H9C	109.5	C25—C26—H26A	109.7
H9A—C9—H9C	109.5	C27—C26—H26A	109.7
H9B—C9—H9C	109.5	C25—C26—H26B	109.7
C15—C10—C11	112.03 (18)	C27—C26—H26B	109.7
C15—C10—S1	109.46 (13)	H26A—C26—H26B	108.2
C11—C10—S1	108.18 (14)	C28—C27—C26	111.15 (17)
C15—C10—H10	109.0	C28—C27—H27A	109.4
C11—C10—H10	109.0	C26—C27—H27A	109.4
S1—C10—H10	109.0	C28—C27—H27B	109.4
C10—C11—C12	109.6 (2)	C26—C27—H27B	109.4
C10—C11—H11A	109.7	H27A—C27—H27B	108.0
C12—C11—H11A	109.7	C29—C28—C27	110.74 (17)
C10—C11—H11B	109.7	C29—C28—H28A	109.5
C12—C11—H11B	109.7	C27—C28—H28A	109.5
H11A—C11—H11B	108.2	C29—C28—H28B	109.5
C13—C12—C11	111.5 (2)	C27—C28—H28B	109.5
C13—C12—H12A	109.3	H28A—C28—H28B	108.1
C11—C12—H12A	109.3	C28—C29—C30	111.53 (17)
C13—C12—H12B	109.3	C28—C29—H29A	109.3
C11—C12—H12B	109.3	C30—C29—H29A	109.3
H12A—C12—H12B	108.0	C28—C29—H29B	109.3
C14—C13—C12	110.6 (2)	C30—C29—H29B	109.3
C14—C13—H13A	109.5	H29A—C29—H29B	108.0
C12—C13—H13A	109.5	C29—C30—C25	109.89 (17)
C14—C13—H13B	109.5	C29—C30—H30A	109.7
C12—C13—H13B	109.5	C25—C30—H30A	109.7
H13A—C13—H13B	108.1	C29—C30—H30B	109.7
C13—C14—C15	111.5 (2)	C25—C30—H30B	109.7
C13—C14—H14A	109.3	H30A—C30—H30B	108.2
			
O4—S2—C25—C26	53.45 (16)	C12—C13—C14—C15	−55.9 (3)
C16—S2—C25—C26	−58.32 (15)	C11—C10—C15—C14	−54.0 (3)
O4—S2—C25—C30	−70.08 (14)	S1—C10—C15—C14	−174.00 (17)
C16—S2—C25—C30	178.15 (12)	C13—C14—C15—C10	54.1 (3)
O2—S1—C1—C8	−144.40 (16)	O4—S2—C16—C23	153.83 (16)
C10—S1—C1—C8	105.31 (17)	C25—S2—C16—C23	−93.78 (17)
O2—S1—C1—C2	34.12 (18)	O4—S2—C16—C17	−23.87 (19)
C10—S1—C1—C2	−76.16 (17)	C25—S2—C16—C17	88.52 (18)
C8—C1—C2—C7	0.3 (2)	C23—C16—C17—C18	−178.8 (2)
S1—C1—C2—C7	−178.40 (14)	S2—C16—C17—C18	−0.8 (3)
C8—C1—C2—C3	179.4 (2)	C23—C16—C17—C22	1.0 (2)
S1—C1—C2—C3	0.6 (3)	S2—C16—C17—C22	178.94 (14)
C7—C2—C3—C4	0.3 (3)	C22—C17—C18—C19	0.7 (3)
C1—C2—C3—C4	−178.63 (19)	C16—C17—C18—C19	−179.6 (2)
C2—C3—C4—C5	−0.3 (3)	C17—C18—C19—C20	0.8 (3)
C2—C3—C4—Cl2	178.79 (13)	C17—C18—C19—Cl1	−179.97 (13)
C3—C4—C5—C6	0.4 (3)	C18—C19—C20—C21	−1.7 (3)
Cl2—C4—C5—C6	−178.68 (16)	Cl1—C19—C20—C21	179.09 (15)
C4—C5—C6—C7	−0.5 (3)	C19—C20—C21—C22	1.0 (3)
C5—C6—C7—O1	179.82 (17)	C20—C21—C22—O3	179.60 (17)
C5—C6—C7—C2	0.5 (3)	C20—C21—C22—C17	0.5 (3)
C8—O1—C7—C6	−178.7 (2)	C23—O3—C22—C21	−179.5 (2)
C8—O1—C7—C2	0.7 (2)	C23—O3—C22—C17	−0.34 (19)
C3—C2—C7—C6	−0.4 (3)	C18—C17—C22—C21	−1.4 (3)
C1—C2—C7—C6	178.8 (2)	C16—C17—C22—C21	178.85 (19)
C3—C2—C7—O1	−179.83 (16)	C18—C17—C22—O3	179.42 (15)
C1—C2—C7—O1	−0.6 (2)	C16—C17—C22—O3	−0.37 (19)
C2—C1—C8—O1	0.0 (2)	C17—C16—C23—O3	−1.2 (2)
S1—C1—C8—O1	178.82 (13)	S2—C16—C23—O3	−179.34 (13)
C2—C1—C8—C9	178.08 (19)	C17—C16—C23—C24	178.90 (19)
S1—C1—C8—C9	−3.1 (3)	S2—C16—C23—C24	0.8 (3)
C7—O1—C8—C1	−0.4 (2)	C22—O3—C23—C16	1.0 (2)
C7—O1—C8—C9	−178.81 (16)	C22—O3—C23—C24	−179.11 (16)
O2—S1—C10—C15	−179.02 (15)	C30—C25—C26—C27	−56.8 (2)
C1—S1—C10—C15	−68.41 (16)	S2—C25—C26—C27	−177.59 (14)
O2—S1—C10—C11	58.67 (17)	C25—C26—C27—C28	57.1 (2)
C1—S1—C10—C11	169.27 (15)	C26—C27—C28—C29	−57.4 (2)
C15—C10—C11—C12	55.3 (3)	C27—C28—C29—C30	56.8 (3)
S1—C10—C11—C12	175.99 (18)	C28—C29—C30—C25	−55.9 (2)
C10—C11—C12—C13	−57.2 (3)	C26—C25—C30—C29	56.2 (2)
C11—C12—C13—C14	58.1 (3)	S2—C25—C30—C29	−178.84 (13)

### Hydrogen-bond geometry (Å, °)

**Table:**

*D*—H···*A*	*D*—H	H···*A*	*D*···*A*	*D*—H···*A*
C5—H5···O2^i^	0.95	2.54	3.469 (3)	166

Symmetry codes: (i) *x*, *y*+1, *z*.
